# Determinants of preeclampsia in women with type 1 diabetes

**DOI:** 10.1007/s00592-017-1053-3

**Published:** 2017-10-03

**Authors:** Paweł Gutaj, Agnieszka Zawiejska, Urszula Mantaj, Ewa Wender-Ożegowska

**Affiliations:** Division of Reproduction, Department of Obstetrics, Gynecology and Gynecological Oncology, Poznan University of Medical Sciences, 33 Polna St, 60-535 Poznań, Poland

**Keywords:** Pregestational diabetes, Type 1 diabetes, Preeclampsia, Gestational hypertension

## Abstract

**Aims:**

Despite improvement in diabetic care over the years, the incidence of hypertensive disorders of pregnancy is still very high. Therefore, the aim of our study was to determine risk factors for PE in women with T1DM.

**Methods:**

This study was a prospective, nested case–control study on a population of 165 women with T1DM. Women were divided into 3 subgroups: normotensive (*N* = 141), gestational hypertension (GH) (*N* = 8), and PE (*N* = 16). Clinical data were collected in the first trimester (< 12th week), in mid-pregnancy (20–24th weeks), and just prior to delivery (34–39th weeks). IR in the first trimester was quantified using the estimated glucose disposal rate formula (eGDR, milligrams/kilogram/minute). Simple logistic regression was used to search for factors associated with PE and GH. For multivariate comparisons, we used multiple logistic regression with stepwise selection.

**Results:**

All preeclampsia cases were diagnosed in primiparae. The presence of vasculopathy was the strongest determinant of PE (OR 10.8, 95% CI 3.27–35.97, *P* = 0.0001), followed by a history of chronic hypertension (6.05, 1.75–20.8, *P* = 0.004) and the duration of diabetes (1.11, 1.03–1.12, *P* = 0.009). However, chronic hypertension and duration of diabetes were no longer associated with PE after adjustment for the presence of vasculopathy. Higher gestational weight gain (GWG) was associated with PE, and this association remained significant after adjustment for first trimester body mass index (1.14, 1.02–1.28, *P* = 0.02). Both systolic and diastolic blood pressure assessed in the first trimester were significant determinants of PE; however, this association was no longer observed after adjustment for the presence of chronic hypertension. Glycated hemoglobin (HbA_1c_) levels from all 3 trimesters were significantly associated with PE (first trimester: 1.38, 1.01–1.87, *P* = 0.04; second trimester: 2.76, 1.43–5.31, *P* = 0.002; third trimester: 2.42, 1.30–4.51, *P* = 0.005). There was a negative association between eGDR and PE (0.66, 0.50–0.87, *P* = 0.003). Among lipids, triglycerides (TG) in all 3 trimesters were positively associated with PE, and this association was independent of HbA_1c_ levels (first trimester: 5.32, 1.65–17.18, *P* = 0.005; second trimester: 2.52, 1.02–6.26, *P* = 0.05; third trimester: 2.28, 1.39–3.74, *P* = 0.001. We did not find any predictors of GH in the regression analysis among all analyzed factors.

**Conclusions:**

Primiparity and diabetic vasculopathy seem to be the strongest risk factors for PE in women with type 1 diabetes. However, preexisting hypertension and higher GWG were also associated with PE in women with T1DM. Among laboratory results, higher HbA_1c_ and TG levels in all 3 trimesters were associated with PE. The association between higher IR and PE in women with T1DM needs further study.

## Introduction

Preeclampsia (PE) is a leading cause of maternal and neonatal morbidity and mortality. It complicates around 3% of pregnancies. There are several risk factors for PE including primiparity, multifetal gestation, maternal age, and pregestational diabetes [1]. The incidence of PE among diabetics ranges from 10 to 20%. Among women with diabetic nephropathy, these numbers are much higher [2]. The pathogenesis of PE is still not fully understood, but it seems to be a disease of placental origin. A diabetic environment as well as preexisting maternal vasculopathy can predispose to reactive oxygen species formation and affect placental function from early pregnancy [3]. Hyperglycemia has been also found to be associated with PE. However, there are some discrepancies between studies regarding what time of action of hyperglycemia is crucial for the development of PE. Temple et al. [4] showed that both preconceptional counseling and first trimester glycated hemoglobin (HbA_1c_) were not associated with PE. Elevated levels of total cholesterol, non-high-density lipoprotein (non-HDL) cholesterol, and triglycerides (TG) during all trimesters of pregnancy, as well as lower levels of high-density lipoprotein (HDL) cholesterol in the third trimester, were associated with PE; however, data from the diabetic population are scarce [5]. The same refers to insulin resistance (IR) and other components of metabolic syndrome [6]. The aim of this study was to establish risk factors for PE in a prospectively recruited cohort of women with type 1 diabetes (T1DM) managed by a single tertiary obstetric center.

## Materials and methods

This prospective study was conducted in the Gynecologic and Obstetrical University Hospital in Poznan, Poland, in a group of 165 pregnant women with T1DM during a period between June 2012 and December 2014. Anthropometric, clinical, and laboratory data were collected during 3 standard hospitalizations that took place in our hospital in the first trimester (< 12th week of gestation-I), in mid-pregnancy (20–24th weeks of gestation-II), and just prior to delivery (34–39th weeks of gestation-III). A detailed description of the study protocol was previously described elsewhere [7, 8]. The therapeutic target in women diagnosed with hypertensive disorders of pregnancy was blood pressure < 135/85 mmHg [9].

Women were divided into 3 subgroups according to pregnancy outcome: normotensive (*N* = 141), developing gestational hypertension (GH) (*N* = 8), and developing PE (*N* = 16).

Blood samples were taken after overnight fasting and immediately transported to the central laboratory of the Gynecologic Obstetrical University Hospital in Poznan for analysis. HbA_1c_ level in whole blood was determined using the turbidimetric inhibition immunoassay (TINIA) (Tina-quant Hemoglobin A1c II test in a Cobas c311 analyzer [Roche Diagnostics, Basel, Switzerland]). The normal range for this test is 4.8–6.0% (29–42 mmol/mol) for a non-pregnant population.

Total serum cholesterol, HDL cholesterol, and TG levels were determined with Roche Diagnostics reagents (Cholesterol CHOD-PAP, HDL-C plus, and Triglycerides GPO-PAP, respectively) on a Cobas c501 analyzer. The following formula was used to calculate the level of low-density lipoprotein (LDL) cholesterol: LDL cholesterol = total cholesterol—HDL cholesterol—(TG/5).

Anthropometric measurements (height, weight, and waist/hip circumference) and blood pressure measurements were performed at the onset of the study. Blood pressure during the first hospitalization was measured 4 times a day and the mean systolic and diastolic value was recorded in the study data.

IR was quantified using the estimated glucose disposal rate (eGDR) (milligrams/kilogram/minute), calculated with the following equation:

$$ {\text{eGDR}} = 24.31 - \left( {12.22 \, \times {\text{WHR}}} \right) \, - \, \left( {3.29 \times {\text{HTN}}} \right) - (0.57 \times {\text{HbA}}_{{ 1 {\text{c}}}} ), $$

where WHR is waist-to-hip ratio, HTN is hypertensive status (0 = no; 1 = yes), and HbA_1c_ is expressed as a percentage. A decrease in eGDR reflects an increase in IR. PE and GH were diagnosed using criteria proposed by the American College of Obstetricians and Gynecologists [10].

Statistical analyses were performed using MedCalc for Windows, version 12.1.3.0 (MedCalc Software, Mariakerke, Belgium). Testing for normality of data distribution was performed using the D’Agostino–Pearson test. The Kruskal–Wallis test was used for intergroup comparisons. The Chi-square test was used for the comparison of categorical variables. Simple logistic regression was used to search for factors associated with PE and GH. For multivariate comparisons, we used multiple logistic regression with stepwise selection. Based on clinical judgment, we included the following confounders into the stepwise models: maternal age, time since diagnosis of diabetes, body mass index (BMI) at onset of the study, parity (multipara vs. primipara), the presence of vasculopathy, and HbA_1c_. Statistical significance was defined as *P* < 0.05 (two sided). The presence of vasculopathy was defined as being diagnosed with at least one of the following: retinopathy, nephropathy, ischemic heart disease. Small for gestational age (SGA) was defined as birth weight lower than 10th percentile, and large for gestational age (LGA) was defined as birth weight greater than 90th percentile using age- and sex-specific regional growth charts [11]. Gestational weight gain (GWG) was categorized based on Institute of Medicine (IOM) 2009 guidelines [12].

The Institutional Ethical Committee of Poznan University of Medical Sciences (No. 673/12, June 12, 2012) approved the study protocol. Informed, written consent was obtained from every patient before inclusion into the study.

## Results

A total of 179 Caucasian women were included in the study. Of those, 14 women were excluded from the analysis due to spontaneous abortions (*N* = 13) and twin pregnancy (*N* = 1), resulting in a total of 165 women included in the final analysis. Of the women included in the final analysis, 16 (9.7%) developed PE, 8 (4.8%) were diagnosed with GH, and 141 did not develop either PE or GH.

The characteristics of the study subgroups are shown in Tables 1and 2. Among the 3 study subgroups, there were no differences in gestational age at the time of study inclusion, maternal age, the proportion of women receiving preconception care, BMI, or WHR. There were no differences in the proportion of women with normal BMI, underweight, and obesity. Proportion of overweight was higher in women who developed PE and PIH than in controls. There was a nonsignificant trend for higher weight gain in women who developed PE. Women who developed PE were diagnosed with diabetes earlier and had a longer duration of the disease than women who were normotensive or had GH. There was significant difference in the proportion of primiparae/multiparae among subgroups. All PE cases were diagnosed in primiparae. Women who developed PE had higher systolic and diastolic blood pressure than controls, but similar to those who developed GH. There was no difference in systolic and diastolic blood pressure between women with GH and controls. Birth weight of newborns born to mothers with PE was lower than in normotensive and GH subgroups; however, the proportions of newborns small for gestational age and sex and large for gestational age and sex were comparable between subgroups.

**Table 1:** Characteristics of the study group

	Control	GH	Preeclampsia	*P*
	(a)	(b)	(c)	
	*N* = 141	*N* = 8	*N* = 16	
Gestational age at onset of study, weeks (mean ± SD)	9 ± 2	10 ± 2	8 ± 2	0.08
Maternal age at onset of study, years (mean ± SD)	29 ± 4	29 ± 7	27 ± 4	0.182
Preconception care (*n* [%])	57 (40.4)	3 (37.5)	3 (18.8)	0.2842
Age at the time of diagnosis of diabetes (mean ± SD)	17 ± 8 c	21 ± 8 c	10 ± 4 a,b	0.001
Duration of diabetes, years (mean ± SD)	12 ± 7 c	12 ± 8 c	17 ± 7 a,b	0.0196
BMI at onset of study, kg/m^2^ (median [IQR])	22.6 (20.7–24.8)	24.1 (23.1–26.6)	24.7 (21.2–26.8)	0.064
Weight distribution (*N*;%): normal weight (BMI 18.5–24.9)	100/141; 70.9%	5/8; 62.5%	8/16; 50%	0.15
Underweight (BMI < 18.5)	6/141; 4.3%	0/8; 0%	1/16; 6.3%	0.15
Overweight (BMI 25.0–29.9)	26/141; 18.4%	3/8; 37.5%	6/16; 37.5%	0.04
Obese (BMI > 30)	9/141; 6.4%	0/8; 0%	1/16; 6.3%	0.55
Waist-to-hip ratio at onset of study (mean ± SD)	0.79 ± 0.06	0.80 ± 0.07	0.76 ± 0.05	0.282
Gestational weight gain, kg (mean ± SD)	11.7 ± 4.7	12.6 ± 5.3	14.7 ± 5.8	0.07
*Gestational weight gain according to Institute of Medicine Guidelines* (*N*; %) [12]				
Normal	59; 41.8%	2; 25%	6; 37.5%	0.96
Lower than recommended	49; 34.8%	3; 37.5%	3; 18.8%	0.60
Higher than recommended	33; 23.4%	3; 37.5%	7; 43.8%	0.09
Primipara/multipara	80/61	5/3	16/0	0.0034
Vasculopathy (cases/group, %)	28/141, 19.9%	1/8, 12.5%	12/16, 75%	0.05
Systolic blood pressure at onset of the study, mmHg (mean ± SD)	115 ± 11 c	120 ± 7	122 ± 15 a	0.026
Diastolic blood pressure at onset of the study, mmHg (mean ± SD)	71 ± 8 c	68 ± 23	76 ± 9 a	0.04
Gestational age at delivery, weeks (median [IQR])	38 (37–39)	38 (37–39)	37 (36–38)	0.08
Birth weight, g (median [IQR])	3540 (3120–3900) c	3780 (3510–4205) c	3040 (2833–3615 a,b	0.048
SGA newborns (cases/group, %)	5/141, 3.5%	0/8 0%	0/16, 0%	0.35
LGA newborns (cases/group, %)	44/141, 31.2%	4/8, 50%	5/16, 31.2%	0.31

Letters a,b,c indicate statistical differences between subgroups (Kruskal–Wallis test)

**Table 2:** Selected laboratory parameters

	Control	GH	Preeclampsia	*P*
	(a)	(b)	(c)	
	*N* = 141	*N* = 8	*N* = 16	
HbA_1c_ I; %; mmol/mol	6.6 (5.9–7.5) c	6.6 (6.3–6.9)	7.8 (6.6–8.9) a	0.048
	49 (41–58)	49 (45–52)	62 (49–7.4)	
HbA_1c_ II; %; mmol/mol	5.6 (5.2–6.0) c	6.0 (5.9–6.4)	6.1 (5.6–6.9) a	0.009
	38 (33–42)	42 (41–46)	43 (38–52)	
HbA_1c_ III; %; mmol/mol	5.8 (5.5–6.3) c	6.2 (5.8–6.7)	6.3 (6.0–7.0)	0.004
	40 (37–45)	44 (40–50)	45 (42–53)	
eGDR; mg/kg/min	10.8 (9.6–11.5) c	11.4 (10.8–11.80 c	10.1 (6.6–11.0) a,b	0.02
TG I; mmol/L	0.65 (0.52–0.83)	0.68 (0.56–0.77)	0.96 (0.58–1.17)	0.1
TG II; mmol/L	1.43 (1.16–1.18)	1.37 (0.96–1.57)	1.65 (1.28–2.28)	0.23
TG III; mmol/L	2.53 (2.03–3.09) c	2.31 (1.91–2.65) c	3.19 (2.55–4.92) a,b	0.017
Creatinine clearance I; ml/min	131 ± 35 c	143 ± 31 c	109 ± 39 a,b	0.04
Daily urinary protein excretion; g/24 h	0.19 (0.09–0.2)	0.15 (0.1–0.27)	0.18 (0.08–0.22)	0.71

Letters a,b,c indicate statistical differences between subgroups (Kruskal–Wallis test)

The results of univariate logistic regression are shown in Table 3.

**Table 3:** Univariate analysis of factors predictive for PE in women with T1DM

Determinants of PE	Crude OR	95% CI	*P*
Duration of diabetes, years	1.11	1.03–1.12	0.009
Vasculopathy (1—yes, 0—no)	10.84	3.27–35.97	0.0001
Chronic hypertension (1—yes, 0—no)	6.05	1.75–20.8	0.004
Gestational weight gain, kg	1.14	1.02–1.28	0.02
Systolic blood pressure at onset of the study, mmHg	1.06	1.01–1.11	0.02
Diastolic blood pressure at onset of the study, mmHg	1.09	1.02–1.16	0.01
HbA1c I; %	1.38	1.01–1.87	0.04
HbA1c II; %	2.76	1.43–5.31	0.02
HbA1c III; %	2.42	1.30–4.51	0.005
eGDR, mg/kg/min	0.66	0.50–0.87	0.003
Triglycerides I; mol/L	5.32	1.65–17.18	0.005
Triglycerides II; mmol/L	2.52	1.02–6.26	0.05
Triglycerides III; mmol/L	2.28	1.39–3.74	0.001

The presence of vasculopathy was the strongest determinant of PE, followed by a history of chronic hypertension and the duration of diabetes. However, chronic hypertension and duration of diabetes were no longer associated with PE after adjustment for the presence of vasculopathy. Higher gestational weight gain (GWG) was associated with PE, and this association remained significant after adjustment for first trimester BMI. Both systolic and diastolic blood pressure assessed in the first trimester were significant determinants of PE; however, this association was no longer observed after adjustment for the presence of chronic hypertension. HbA_1c_ levels from all 3 trimesters were significantly associated with PE. There was a negative association between eGDR and PE. Among lipids, TG level in all 3 trimesters was positively associated with PE, and this association was independent of HbA_1c_ levels. There was no association between maternal age, BMI, WHR, preconception care, HDL, LDL, total cholesterol, or 24-h urine protein test and PE. We did not find any predictors of GH in the regression analysis among all analyzed factors.

## Discussion

The aim of our study was to search for factors associated with PE and GH in women with T1DM. All women included in this prospective, observational study were managed in a single obstetric center for women with diabetes. It gave us the possibility to collect a wide range of clinical and laboratory data from the entire duration of pregnancy. We showed that many factors may be involved in the pathogenesis of PE in women with T1DM; however, their individual predictive value is relatively low. Interestingly, GH had no predictors in our study group. This might be at least partly explained by the small number of patients in the GH subgroup which could have interfered with statistical power of the analysis. However, these discrepancies may also reflect the different pathogenesis of GH and PE. Some authors suggest that GH and PE are independent diseases. In their large epidemiological study, Ros et al. [13] demonstrated that T1DM was a significant risk factor for PE, but not for GH. In our study, the incidence of PE was 2 times higher than GH, which seems to confirm the results presented by Ros et al.

Valuable data supporting differences in GH and PE etiology come from studies on maternal hemodynamics and angiogenic biomarkers. Noori et al. [14] demonstrated that women with GH had hyperdynamic circulation and an angiogenic biomarker profile (placental growth factor [PLGF] and soluble fms-like tyrosine kinase-1 [sFlt-1]) similar to normotensive pregnant controls, while these factors were significantly altered in women with PE. Moreover, mothers with PE (but not with GH) showed venous hemodynamic abnormalities [15]. It has been shown that diabetes, especially when longstanding and complicated by vascular disease, can lead to placental dysfunction, similar to that observed in PE and manifested by changes in both angiogenic biomarkers and restricted fetal growth [16]. In the present study, longer diabetes duration and diabetic vascular disease were associated with PE but not GH. However, diabetes duration was no longer associated with PE after adjustment for the presence of vasculopathy. This indicates that excellent glycemic control as a main method of prevention of diabetic vascular disease can also prevent women from developing PE and its associated complications. Chronic hypertension has been found to predispose to PE [17]. We also found this association in our study; however, it was no longer observed after adjustment for vasculopathy. The effect of hypertension on PE risk might be time dependent, as shown by Sibai et al. In their large, multicenter study, the authors demonstrated that only a history of hypertension lasting for at least 4 years was associated with a higher rate of PE. Due to a relatively small number of patients, we were not able to address such effects in our population. Nonetheless, in a population of T1DM patients, the incidence of hypertension is strongly associated with the presence of diabetic nephropathy, which usually develops many years after the onset of diabetes [18]. Moreover, in the same study, the incidence of hypertension among diabetics with normal urinary protein excretion was similar to that observed in the general population. This suggests that hypertension in this population is rather the consequence of kidney disease. Based on our results, we can also conclude that it is vasculopathy and not hypertension itself that is probably responsible for placental dysfunction and subsequent PE. Intensified antihypertensive treatment in women with diabetic nephropathy, together with aspirin, could probably reduce the rates of PE in this high-risk population [9].

Interestingly, there was no association between increased BMI in the first trimester and PE in our population. Such an association was demonstrated in previous studies and confirmed in a recent meta-analysis [19]. The lack of association between BMI and PE in our study might be explained by the small number of women with overweight and obesity. In a recent population-based cohort study among diabetic women, Persson et al. [20] demonstrated that risk of PE was independent of BMI, but the odds for PE increased further with maternal overweight and obesity among women with T1DM. Nonetheless, the effect of maternal weight on PE risk was rather weak in women with T1DM. According to the authors, this might be explained by the fact that T1DM is a much stronger risk factor for PE than BMI.

Additionally, GWG up to 18 weeks of pregnancy, independent of prepregnancy weight, has been found to increase risk of hypertensive disorders of pregnancy in a general population [21]. In our study, we analyzed GWG over the entire course of pregnancy, but we showed similar results. This suggests that weight control during pregnancy in women with T1DM may also have beneficial effects on the incidence of PE.

Primiparity is a major risk factor for PE. In our study, all PE cases were diagnosed in primiparae. Such disproportion was not observed among controls or the GH subgroup where parity status was comparable. This suggests that primiparity also seems to be a risk factor for PE among women with T1DM; however, such an association was not observed in some studies [22, 23].

Excellent glycemic control is crucial for normal fetal development during pregnancy. We showed that HbA_1c_ levels in all trimesters were positively related to the odds of developing PE, which is in line with the results of previous studies [4, 23–25]. Similarly to the study published by Temple et al., there was no effect of attending preconception care and the incidence of PE in our cohort [4]. However, we found an association between first trimester HbA_1c_ and PE risk, which is in line with the results of the majority of studies [23–25], but not with the results of Temple et al., who did not show such an association. Bearing in mind the pathogenesis of PE, which is believed to have its roots early in pregnancy, hyperglycemia during that period can negatively impact placental structure and function [26, 27]. Therefore, we believe that strict glycemic control starting from the very first week of pregnancy should be considered for primary prevention of PE in women with T1DM.

Increased IR, other parameter representing oxidative stress has been shown to predispose non-diabetic women and those with gestational diabetes to PE [28]. To the authors’ knowledge, this is the first study that evaluated IR in early pregnancy in women with T1DM. For this purpose, we used the eGDR formula—a noninvasive method of IR quantification standardized for patients with T1DM which closely correlates with the gold standard method (glucose clamp technique). eGDR values are inversely correlated with IR. We demonstrated that eGDR was negatively associated with PE. The other novel finding of our study is the positive association between TG concentration in all 3 trimesters and PE. A similar association was shown in previous studies; however, it was carried out mostly among non-diabetics [29]. Increased IR and hypertriglyceridemia are typical features of metabolic syndrome, and there is a need for interventional trials to determine whether prepregnancy weight reduction and dietary intervention could lower the risk of PE in women with T1DM.

PE has been shown to predispose to intrauterine growth restriction. Interestingly there were no newborns with SGA in both PE and PIH groups. For the purpose of this study, we used percentile charts for general population. Pearson et al. demonstrated that birth weight distribution of offspring born to women with type 1 diabetes is shifted to the right of the normal reference [30]. It may be hypothesized that some growth-restricted fetuses of the diabetic mothers can be falsely “normalized” by maternal hyperglycemia. This could have been possible in our population of women with PE as their metabolic control was worse than in controls.

Our study has limitations. Like in other prospective studies, the number of women who developed PE and GH was small. Moreover, we did not include data on aspirin use in the analysis. However, until 2015, there were no recommendations for such prophylaxis in women with pregestational diabetes in Poland; therefore, it was very rarely used by pregnant women at the time of the study.

The strength of the current study is that all women were managed in a single center according to the same protocols. Because all women with T1DM admitted to our department during the study period were recruited to the study and we used a nested case–control design, selection bias was minimized. Furthermore, the study period was relatively short, and we used comparable therapeutic targets throughout the study. Moreover, all laboratory measurements were performed in the same certified laboratory, thus limiting the possibility of non-random errors resulting from procedural differences. Finally, because of the prospective design, we were able to collect a wide range of clinical and laboratory data including estimation of eGDR, which is novel in the literature.

To summarize, we demonstrated that the incidence of PE among women with T1DM remains high and many factors might be involved in its pathogenesis. However, there is a need for further research in this area, especially aimed at searching for effective methods for prevention of PE, such as dietary interventions and prepregnancy weight control to reduce IR in early pregnancy.
